# Regional population decline and health screening uptake in Korean adults: nationwide study using multilevel regression analysis

**DOI:** 10.3389/fpubh.2025.1507691

**Published:** 2025-04-25

**Authors:** Wonjeong Jeong, Woorim Kim, Kyu-Tae Han

**Affiliations:** ^1^Cancer Knowledge and Information Center, National Cancer Control Institute, National Cancer Center, Goyang, Republic of Korea; ^2^National Hospice Center, National Cancer Control Institute, National Cancer Center, Goyang, Republic of Korea; ^3^Division of Cancer Control and Policy, National Cancer Control Institute, National Cancer Center, Goyang, Republic of Korea

**Keywords:** regional population change, health screening, cancer screening, medical accessibility, regional disparity

## Abstract

**Background:**

Health screening is crucial for detecting medical needs and presenting effective alternatives. As Korea undergoes rapid demographic shifts and widening regional gaps, screening is increasingly important to identify these needs. This study explores how changes in regional population size related to health screening uptake among Korean adults.

**Methods:**

Data on 182,437 adults from the 2021 Korean Community Health Survey (KCHS) were used, with health screening divided into cancer and general medical screening. Regional population size, aging index and financial independence ratio from 2012 to 2022 KOSIS were linked to our data. Generalized linear mixed effects models were applied for hierarchical logistic regression analysis of the association between the regional population size and screening, controlling for regional- and individual-level variables.

**Results:**

Decrease in regional population size were significantly associated with lower odds ratio (OR) of receiving health screening; OR 0.85 (95% CI 0.83–0.88), as well as cancer screening; OR 0.87 (95% CI 0.85–0.90). Similar results were observed in regions with stable in population size.

**Conclusion:**

Our study findings indicate the significant associations between regional population size decline and screening. Population-based policies should consider regional attributes to ensure equitable access to screening services.

## Background

Health screening has long been regarded as a primary preventative approach for incidence and progression of disease (1). In Korea, non-communicable diseases such as cancer have consistently ranked among the top causes of death for decades (2). As of 2022, the total number of prevalent cancer cases in South Korea has exceeded 2 million since 2018, indicating that approximately one in in 20 individuals has a history of cancer diagnosis (3, 4). Moreover, the five most common type of cancer are projected to account for 55.7% of the total cancer burden in Korea, a figure expected to rise as the population continues to age (5). To mitigate the burden of disease, health screening in Korea is offered through a variety of organized and opportunistic screening programs (6). National efforts to combat disease include the introduction of the General Health Screening Program, which offers screening services at little to no cost to eligible individuals. Additionally, the Korean government launched the National Cancer Screening Program to address cancer-related mortality and its associated burdens (7, 8).

The healthcare costs are naturally expected to increase with aging, as risk of disease and geriatric conditions is increased exponentially (9, 10). Korea has crossed the threshold criteria of an aged society with a proportion of older adults of 17.5% in 2022, which could be attributed to an increase in life expectancy and a record-low in birth rates (11). In this context, timely screening becomes paramount due to the disappearance of regions caused by aging, decreasing fertility rates, and deepening regional gaps. These demographic shifts, such as population aging and declining fertility rates, have become primary drivers of changes in population dynamics and resultant population decline, leading to local extinction (12). In particular, it has been previously reported that many regions in Korea are currently experiencing rapid depopulation, with some on the verge of extinction, i.e., “shrinking cities” (13). This phenomenon is not confined solely to Korea; similar patterns have been observed in nearby Japan and China, as well as in certain regions across Europe and the US (14, 15).

Due to the government’s concerns about regional extinction, various healthcare policies have been introduced and are being promoted to close the gap between regions. In 2022, the Korean government put forth a plan to tackle this issue by designating and providing financial support to a total of 90 regions known as “depopulation areas.” Additionally, in terms of essential health services, policies are now being developed to address the medical imbalance between regions (16). Nonetheless, although existing literature suggests that the regional population decline may compromise the medical infrastructure and access to healthcare services, it is still unclear how health screening uptake due to depopulation may be impacted in the Korean setting (16, 17).

Moreover, a number of studies have reported on relevant individual-level factors to health screening, but impact of regional-level determinants such as population size change on screening is under- researched (18, 19). Growing evidence also points toward the importance of the incorporation of multilevel modeling strategies when identifying screening barriers (19). Therefore, our study’s main objective was to shed light on the effects of regional population change on screening among adults in Korea using a multilevel modeling approach.

## Methods

### Participants

This study utilized data from the 2021 Korean Community Health Survey (KCHS), a nationwide health interview survey conducted by the Korean Centers for Disease Control and Prevention. The primary objectives of the KCHS are to establish and evaluate regional health plans, standardize the survey methodologies, and generate comparable regional health statistics (20, 21). This study included only participants aged 20 years and older and excluded those with missing data on variables on household income level, smoking status, region. A total of 46,566 individuals were excluded because they either considered the information sensitive or reported not knowing the answers. Consequently, a total of 182,437 individuals were included in the final analysis. Regional variables were obtained from the Korean Statistical Information Service (KOSIS) and were used to link each individual to their respective regional code.

#### Variables

The dependent variable of this study was health screening, which included both cancer screening and general medical checkups. Participants were categorized based on their response to the question: ‘Did you undergo general checkups and a cancer screening to assess your health status, even in the absence of specific health problems?’ Additionally, cancer screening were analyzed separately for a more detailed examination.

Population size changes in each region were calculated by dividing the 2022 population by the 2012 population, using data from KOSIS. These changes were treated as a continuous variable and then categorized into three groups: increase (greater than 0%), stable (0–10% reduction), or decrease (greater than 10% reduction). The 2022 data represented the most recent population statistics available, while 2012 marked a year of significant geographic changes, including the establishment of Sejong city as a self-governing province, making it a suitable reference point for a 10-year analysis. Additionally, as policies aimed at expanding health insurance coverage to improve patient access concluded in December 2009, this timeframe was appropriate for evaluating subsequent changes in public health conditions.

Region-related variables, including financial independence and the aging index, were obtained from the KOSIS and linked with each individual’s regional codes of residence. These variables were measured using the combined regional codes, and median values for the low and high categories were calculated using the data from KOSIS. Regional financial independence ratio has shown that the capability level of an area to self-financing the government activities, development and provide the good service to people who paid off the taxes and levies as source of income whom needed by the region (22). Aging index is the age of a society, which is the ratio of those aged 65 and over against those aged 0–14 (23).

The individual-level characteristics controlled for in this study included age, sex, marital status, household income level, region, alcohol consumption status, smoking status, self-reported health status, and health literacy. Health literacy encompassed the ability to understand verbal health information, such as verbal explanation by clinicians, as well as the ability to comprehend written health information, such as that found on the internet or in brochure.

### Statistical analysis

Chi-square tests were conducted to examine the general characteristics of the study population. Generalized linear mixed models (PROC GLIMMIX) were employed for hierarchical logistic regression analysis to investigate the association between regional population size and screening. A multilevel model is a special case of generalized linear mixed models that can be handled by the GLIMMIX procedure (24). These multilevel models were used to account for potential correlations within the same region (25). The initial model included individuals-level variables to access their impact on screening. Model 2 focused on the influence of regional-level variables, with the region included as a random effect to explore its unique contributions. Finally, the last model (model 3) incorporated both individuals and regional-level variables. Intraclass correlations (ICC) were used to evaluate whether there was significant variation between groups compared to variation within those groups (26). ICC is calculated as the ratio of the variance between clusters to the total variance. Results are presented as odds ratio (OR) and confidence intervals (CI). Statistical significance was determined at *p*-value < 0.05. All data analyses used SAS 9.4 software.

## Results

Table 1 describes the general characteristics of the study participants. Among 182,437 respondents, 105,460 individuals (or 57.8% of the total study sample) reported to have undergone health screening. In terms of regional-level characteristics, those who reported to have undergone health screening for regional population size were shown as follows: 56.5, 58.4 and 58.5% of participants in the ‘decrease’, ‘stable’ and ‘increase’ groups, respectively. For the aging index, those who reported to have undergone screening were 57.2% in the ‘low’ vs. 58.4% of individuals in the ‘high’ group. Additionally, 60.0% vs. 55.6% of participants in the ‘low’ and ‘high’ groups of the financial independence ratio reported to have undergone health screening.

**Table 1:** General characteristics of the study population.

Variables	Total		Health screening				*p*-value
			Yes		No		
	*N*	(%)	*N*	(%)	*N*	(%)	
Regional-level characteristics							
Regional population size							<0.0001
Decrease (≥ 10% reduction)	60,633	(33.2)	34,272	(56.5)	26,361	(43.5)	
Stable (0–10% reduction)	69,002	(37.8)	40,319	(58.4)	28,683	(41.6)	
Increase (> 0%)	52,802	(28.9)	30,869	(58.5)	21,933	(41.5)	
Aging index							<0.0001
Low	87,462	(47.9)	50,010	(57.2)	37,452	(42.8)	
High	94,975	(52.1)	55,450	(58.4)	39,525	(41.6)	
Financial independence ratio							<0.0001
Low	90,752	(49.7)	54,491	(60.0)	36,261	(40.0)	
High	91,685	(50.3)	50,969	(55.6)	40,716	(44.4)	
Individual-level characteristics							
Age (years)							<0.0001
19–39	39,302	(21.5)	9,698	(24.7)	29,604	(75.3)	
40–49	26,747	(14.7)	16,902	(63.2)	9,845	(36.8)	
50–59	33,316	(18.3)	23,028	(69.1)	10,288	(30.9)	
60–69	37,979	(20.8)	27,300	(71.9)	10,679	(28.1)	
≥ 70	45,093	(24.7)	28,532	(63.3)	16,561	(36.7)	
Sex							<0.0001
Male	81,446	(44.6)	44,462	(54.6)	36,984	(45.4)	
Female	100,991	(55.4)	60,998	(60.4)	39,993	(39.6)	
Marital status							<0.0001
Married	111,799	(61.3)	75,083	(67.2)	36,716	(32.8)	
Separated or divorced	38,769	(21.3)	23,257	(60.0)	15,512	(40.0)	
Unmarried	31,869	(17.5)	7,120	(22.3)	24,749	(77.7)	
Household income level							<0.0001
Low	29,693	(16.3)	16,474	(55.5)	13,219	(44.5)	
Middle	83,166	(45.6)	48,849	(58.7)	34,317	(41.3)	
High	69,578	(38.1)	40,137	(57.7)	29,441	(42.3)	
Region							<0.0001
Metropolitan	53,177	(29.1)	30,093	(56.6)	23,084	(43.4)	
City	38,887	(21.3)	20,325	(52.3)	18,562	(47.7)	
Rural	90,373	(49.5)	55,042	(60.9)	35,331	(39.1)	
Alcohol status							<0.0001
Never	42,635	(23.4)	25,964	(60.9)	16,671	(39.1)	
Ever	139,802	(76.6)	79,496	(56.9)	60,306	(43.1)	
Smoking status							<0.0001
Never	119,417	(65.5)	69,971	(58.6)	49,446	(41.4)	
Ever	63,020	(34.5)	35,489	(56.3)	27,531	(43.7)	
Self-reported health status							<0.0001
High	72,730	(39.9)	39,760	(54.7)	32,970	(45.3)	
Middle	77,280	(42.4)	46,585	(60.3)	30,695	(39.7)	
Low	32,427	(17.8)	19,115	(58.9)	13,312	(41.1)	
Health literacy							<0.0001
Low	75,274	(41.3)	42,888	(57.0)	32,386	(43.0)	
High	107,163	(58.7)	62,572	(58.4)	44,591	(41.6)	
Total	182,437	(100.0)	105,460	(57.8)	76,977	(42.2)	

The multilevel model analysis results for regional population size and health screening are shown in Table 2. We presented results on model 3 as its corresponding goodness-of-fit values indicated the best model fit. In model 3, compared to an increase in the regional population size: stable and decrease in regional population size reported lower odds of health screening: OR 0.95 (95% CI 0.92—0.98) and OR 0.85 (95% CI 0.82—0.88). Compared to a low financial independence ratio, a high ratio was associated with lower odds of health screening: OR 0.93 (95% CI 0.90—0.95).

**Table 2:** Results of regional population size and health screening.

Variables						Health screening						
		Model 1				Model 2				Model 3^a^		
		OR (95% CI)				OR (95% CI)				OR (95% CI)		
Fixed effects intercept (S.E)		0.39*(0.05)				0.94*(0.09)				1.09*(0.08)		
Regional-level characteristics												
Regional population size												
Decrease (≥ 10% reduction)	0.88	(0.85)	–	(0.90)					0.85	(0.82)	–	(0.88)
Stable (0–10% reduction)	0.96	(0.94)	–	(0.99)					0.95	(0.92)	–	(0.98)
Increase (> 0%)	1.00								1.00			
Aging index												
Low	1.00								1.00			
High	1.09	(0.94)	–	(1.27)					0.93	(0.84)	–	(1.04)
Financial independence ratio												
Low	1.00								1.00			
High	0.87	(0.84)	–	(0.89)					0.93	(0.90)	–	(0.95)
Individual-level characteristics												
Age (years)												
19–39					0.28	(0.27)	–	(0.29)	0.28	(0.27)	–	(0.29)
40–49					1.00				1.00			
50–59					1.30	(1.25)	–	(1.35)	1.30	(1.26)	–	(1.35)
60–69					1.64	(1.58)	–	(1.70)	1.64	(1.58)	–	(1.70)
≥ 70					1.40	(1.34)	–	(1.45)	1.40	(1.35)	–	(1.45)
Sex												
Male					0.80	(0.78)	–	(0.83)	0.80	(0.78)	–	(0.83)
Female					1.00				1.00			
Marital status												
Married					1.00				1.00			
Separated or divorced					0.70	(0.68)	–	(0.72)	0.70	(0.68)	–	(0.72)
Unmarried					0.36	(0.35)	–	(0.37)	0.36	(0.35)	–	(0.37)
Household income level												
Low					0.61	(0.59)	–	(0.64)	0.61	(0.59)	–	(0.64)
Middle					0.83	(0.81)	–	(0.85)	0.83	(0.81)	–	(0.85)
High					1.00				1.00			
Region												
Metropolitan					1.00				1.00			
City					0.83	(0.68)	–	(1.01)	0.81	(0.69)	–	(0.96)
Rural					1.08	(0.89)	–	(1.30)	1.06	(0.91)	–	(1.24)
Alcohol status												
Never					1.00				1.00			
Ever					1.15	(1.12)	–	(1.18)	1.15	(1.12)	–	(1.18)
Smoking status												
Never					1.00				1.00			
Ever					0.89	(0.87)	-	(0.92)	0.89	(0.86)	-	(0.92)
Self-reported health status												
High					1.00				1.00			
Middle					1.03	(1.01)	–	(1.05)	1.03	(1.01)	–	(1.06)
Low					0.89	(0.86)	–	(0.92)	0.90	(0.87)	–	(0.92)
Health Literacy												
Low					0.78	(0.76)	–	(0.80)	0.78	(0.76)	–	(0.80)
High					1.00				1.00			
*Error variance*												
Level-2 intercept (S.E)		0.01*(0.02)				0.007*(0.015)				0.004*(0.01)		
*Model fit*												
-2LL		247046.7				216690.7				216555.9		
Pearson Chi-Square/DF		1.00				1.00				1.00		

*p < 0.05; ICC (Intraclass correlation coefficient): 0.0500 (<0.0001). ^a^Best fitting model.

Table 3 presents the results of multilevel analysis of regional population size and cancer screening. Model 3 (best fitting model) results for regional-level characteristics were as follows; for regional population size; stable: OR 0.96 (95% CI 0.94—0.99) and decrease in regional population size: OR 0.87 (95% CI 0.85—0.90) were associated with a lower odds ratio of cancer screening, compared to the increase group. Furthermore, a high financial independence ratio showed decreased odds OR 0.94 (95% CI 0.91—0.96) of cancer screening as compared to low financial independence.

**Table 3:** Results of regional population size and cancer screening.

Variables					Cancer screening							
		Model 1				Model 2				Model 3^a^		
		OR (95% CI)				OR (95% CI)				OR (95% CI)		
Fixed effects												
Intercept (S.E)		0.44*(0.05)				1.03*(0.09)				1.16*(0.08)		
Regional-level characteristics												
Regional population size												
Decrease (≥ 10% reduction)	0.89	(0.87)	–	(0.92)					0.87	(0.85)	–	(0.90)
Stable (0–10% reduction)	0.97	(0.94)	–	(1.00)					0.96	(0.94)	–	(0.99)
Increase (> 0%)	1.00								1.00			
Aging index												
Low	1.00								1.00			
High	1.10	(0.94)	–	(1.28)					0.93	(0.83)	–	(1.05)
Financial independence ratio												
Low	1.00								1.00			
High	0.87	(0.85)	–	(0.90)					0.94	(0.91)	–	(0.96)
Individual-level characteristics												
Age (years)												
19–39					0.29	(0.28)	–	(0.30)	0.29	(0.28)	–	(0.30)
40–49					1.00				1.00			
50–59					1.28	(1.24)	–	(1.33)	1.28	(1.24)	–	(1.33)
60–69					1.62	(1.56)	–	(1.68)	1.62	(1.57)	–	(1.69)
≥ 70					1.35	(1.30)	–	(1.41)	1.35	(1.30)	–	(1.41)
Sex												
Male					0.75	(0.73)	–	(0.78)	0.75	(0.73)	–	(0.78)
Female					1.00				1.00			
Marital status												
Married					1.00				1.00			
Separated or divorced					0.69	(0.67)	–	(0.71)	0.69	(0.67)	–	(0.71)
Unmarried					0.34	(0.33)	–	(0.35)	0.34	(0.33)	–	(0.35)
Household income level												
Low					0.63	(0.61)	–	(0.65)	0.63	(0.61)	–	(0.66)
Middle					0.85	(0.82)	–	(0.87)	0.85	(0.82)	–	(0.87)
High					1.00				1.00			
Region												
Metropolitan					1.00				1.00			
City					0.84	(0.68)	–	(1.03)	0.82	(0.69)	–	(0.98)
Rural					1.08	(0.89)	–	(1.32)	1.07	(0.91)	–	(1.27)
Alcohol status												
Never					1.00				1.00			
Ever					1.15	(1.12)	–	(1.18)	1.15	(1.12)	–	(1.18)
Smoking status												
Never					1.00				1.00			
Ever					0.91	(0.88)	–	(0.94)	0.91	(0.88)	–	(0.94)
Self-reported health status												
High					1.00				1.00			
Middle					1.04	(1.01)	–	(1.06)	1.04	(1.01)	–	(1.06)
Low					0.90	(0.87)	–	(0.93)	0.90	(0.87)	–	(0.93)
Health literacy												
Low					0.78	(0.76)	–	(0.79)	0.78	(0.76)	–	(0.79)
High					1.00				1.00			
*Error variance*												
Level-2 intercept (S.E)		0.011*(0.03)				0.007*(0.02)				0.005*(0.01)		
*Model fit*												
-2LL		245070.4				214561.6				214467.1		
Pearson Chi-Square/DF		1.00				1.00				1.00		

*p < 0.05; ICC (Intraclass correlation coefficient): 0.0500 (<0.0001). ^a^Best fitting model.

## Discussion

Our present study’s results showed that a decrease in regional population size was significantly associated with the lowest likelihood of health screening. The findings also indicated a similar pattern for cancer screening in relation to regional population changes. Regional population decline, driven by aging, declining fertility rates, and migration, reduces access to medical care and challenges service quality, necessitating targeted interventions.

Recent changes in regional population size in 2022, compared to 2012 may be indicative of a variety of drivers including regional fluctuations in birth and mortality rates. Furthermore, a decrease in local population size due to youth out-migration is also an issue of great concern (27). Motivations behind inter-regional migration of young residents are numerous, including the pursuit of improved education, employment opportunities and overall quality of life in other (often more urbanized) regions (28). In 2022, 44.7% of Korea’s total population resided in the capital city, Seoul, and its surrounding metropolitan areas (29). As such, overcrowding in urban areas and depopulation in rural areas may lead to an imbalance and eventual collapse in the medical infrastructure and access to healthcare services (30).

Changes in population size, such as shrinkage, and shifts in population structure, including aging, present significant challenges for many countries. Rural shrinkage, characterized by a sharply declining and increasingly aging population, is a widespread global phenomenon (31). Understanding how different countries manage these demographic shifts is crucial, as many are experiencing similar post-growth trajectories. For instance, in Taiwan, the share of the population aged 65 and over was just 8.4% in 2000 but had nearly double to 16.0% by 2020 (32). In 2021, the proportion of the population aged 65 and older was 28.9% in Japan, 16.6% in South Korea, and 14.2% in China (31). Given that many countries have already entered or are on the brink of population decline, comprehensive investigations into its impact are of considerable significance (33).

As a consequence of an interplay of these factors, most depopulated areas are predominantly inhabited by older adults (30). Aging populations face numerous challenges, including a higher burden of chronic disease and limitations in daily activities, which, in turn, increase the demand for expanded screening services (34, 35). Ensuring adequate screening resources for this vulnerable population is particularly crucial for time-sensitive conditions such as cancer (36). Given these considerations, the inclusion of the aging index was expected to provide valuable insight in our study; however, no statistically significant associations were observed with any type of screening. Previous study shown that older adults are more likely to undergo health checkups, with cancer screening participation rates peaking among individuals aged 60–69 years (37, 38).

In 2022, the Korean government designated approximately 90 regions as ‘depopulation area’ and established one trillion won annual fund to address local extinction risk (16). To address regional health challenges, the government continues to develop strategies across various sectors, including healthcare. This study calculated the regional extinction index by analyzing local population changes to capture both population inflow and outflow. This index serves as a representative measure of population change (39). Unlike previous studies that primarily focused on economically active populations, our approach provides a more comprehensive understanding of overall population dynamics (40). Give that population decline is a significant national concern, it is essential to examine regional population circulation structures, including both inflow and outflow, to inform effective policy responses (16).

From a population perspective, regional-level financial independence reflects the ability of local governments to maintain financial independence. The degree of financial independence of local governments is closely linked to the demographic factors such as an aging population and low birth rates (41). In our study, a high financial independence ratio was found to be inversely associated with health screening attendance. This finding contrasts with the results of Park et al., who, using the 2017 KCHS data, examined the relationship between individual and regional factors and health screening participation. In contrast to our study, they reported no significant associations between financial independence and health screening participation (42). Although the financial independence ratio may not fully represent the overall financial condition of a local government, it remains a key indicator of its fiscal health (41). Therefore, a low financial independence ratio, coupled with unstable demographic trends, could serve as valuable evidence to inform active management and policy interventions aimed at bolstering healthcare infrastructure.

As the population decreases, tax revenue naturally diminishes, which can adversely affect the financial independence ratio. Even with a reduced population, municipalities are required to maintain the same infrastructure network. However, the shrinking tax base may result in higher tax rates or insufficient revenue, ultimately leading to a deterioration in the quality of public services. Furthermore, reductions in gross product and consumption may occur, potentially leading to cuts in essential infrastructure, including health services (43). These demographic shifts are expected to have broad societal implications, including a contraction of labor markets, employment medical examination increased tax burdens to sustain pension systems, and economic stagnations (44). Therefore, proactive government intervention is essential to ensure continued access to infrastructure for residents in areas experiencing population decline.

At the individual-level, health literacy emerged as a significant risk factor for screening participation in our study, irrespective of the type of screening. Limited health literacy is a barrier that may negatively impact screening by affecting the extent to which health information is assimilated, thereby de-empathizing the importance of seeking screening services (45). Given that inadequate health literacy appears to impact Korean older adults more often than their younger counterparts, we believe that focusing on enhancing health literacy could yield additional advantages when customizing relevant health policies (46). In addition to expanding local infrastructure, improving health literacy and implementing public education and awareness campaigns are essential strategies for promoting preventive health behaviors, including participation in health screening programs among local populations.

Limitations were present in our study. First, due to the cross-sectional nature of the data, causal relationships could not be established. Therefore, we cannot determine a causal relationship between regional population decline and health screening. Furthermore, despite efforts by the surveying agency to minimize bias, the data used in this study were primarily self-reported, making them susceptible to potential recall bias. Third, although we adjusted for various regional- and individual-level covariates that could influence the results, we cannot entirely rule out residual confounding, as some unmeasured or unconsidered factors may still exist. Lastly, this study may not fully capture individual-level variations within regions. While regional-level characteristics provide valuable insights into broad trends, individuals variation within these regions may not be fully accounted for.

Nonetheless, there were also some notable strengths. To the best of our knowledge, our study is one of the very first population-based studies to examine the influence of regional population change on health screening using a multilevel modeling approach. We included measures that reflect a region’s population structure and economic status such as the aging index and the financial independence ratio, which are readily available by the KOSIS. The current study also has the advantage of incorporating a large, nationally representative sample of Korean adults.

## Conclusion

Decrease in regional population size was found to show the lowest significant odds with all types of screening. Regional-level intervention programs targeted at growing screening rates may prove effective, on the condition that unique characteristics of the regions including population demographics and size are taken in account.

## Data Availability

The original contributions presented in the study are included in the article/supplementary material, further inquiries can be directed to the corresponding author/s.
